# Computer processable classification of craniofacial clefts

**DOI:** 10.3389/fphys.2015.00067

**Published:** 2015-03-06

**Authors:** Rakesh Koul

**Affiliations:** Department of Orthodontics and Dentofacial Orthopedics, Career Post Graduate Institute of Dental SciencesLucknow, India

**Keywords:** classification of cleft of lip and palate, nasomedial process, antenasal segment, electronic data registration, computer processable

Different interpretations of the term “Cleft Lip” by clinicians and developmental biologists referred to by Wang et al. (2014) in the introduction of their article on evaluation and integration of disparate classification systems for clefts of the lip, mirrors my observations in an article on nomenclature in craniofacial embryology (Koul, 2006).

In that article, I had proposed a change of terminology of pre-palate/primary palate/anterior palate to *Antenasal Segment*, to remove the ambiguity of interpretations of differing terms for components that are involved in “Cleft Lip” formation. This *antenasal* segment comprises of (1) antenasal labium, (2) antenasal gnathia, (3) and antenasal palate in the completed face. This new terminology accurately identifies the embryological origin (naso-medial process) and structural precedence of this segment to the nasal frame of reference. The term *antenasal segment* reconciles the pre-natal and post-natal terminologies, facilitating a correct interpretation of the term “Cleft Lip” for classification and registration of the clefts of this segment in a manner that would reflect its etiologic and phenotypic variability.

Evaluation and integration of various classification systems of “Cleft Lip” by Wang et al. is an important contribution to the process of making informed decisions for a cleft lip patient or a group of patients. The ontology of craniofacial development and malformation (OCDM) being developed as a part of NIDCR funded research network, Facebase will facilitate integration of such data for translational craniofacial research (Brinkley et al., 2013; Mejino et al., 2013). A standardized and uniform recording and retrieval of patient data is of fundamental importance for permitting interoperability with multiple resources in an efficient and effective manner. Toward this end, the classification schemes of cleft lip and palate need to be evaluated not only for their accuracy but also for optimization with electronic data registration and retrieval for multiple applications. With the introduction of medical information technology in clinical medicine, a global computerized classification system for cleft lip and palate is a prerequisite for achieving standardization and uniformity.

Koul's classification of cleft lip and palate (Koul, 2007) expresses clinical data with a universally accepted natural language that corresponds to the nomenclature of the affected structures of the clefts. The use of this natural language in upper and lower case formats in combination with other keyboard signs and symbols makes this system of classification computer- processable. These are interpretation of facts, further verification and comparison studies are required to arrive at definitive conclusions. A universal, computerized classification system of cleft lip and palate will facilitate efficient and effective recording and retrieval of craniofacial data for translational research.

## Conflict of interest statement

The author declares that the research was conducted in the absence of any commercial or financial relationships that could be construed as a potential conflict of interest.
